# Myelitis features and outcomes in CNS demyelinating disorders: Comparison between multiple sclerosis, MOGAD, and AQP4-IgG-positive NMOSD

**DOI:** 10.3389/fneur.2022.1011579

**Published:** 2022-11-07

**Authors:** Giulia Fadda, Eoin P. Flanagan, Laura Cacciaguerra, Jiraporn Jitprapaikulsan, Paolo Solla, Pietro Zara, Elia Sechi

**Affiliations:** ^1^Montreal Neurological Institute, McGill University, Montreal, QC, Canada; ^2^Department of Neurology, Center for MS and Autoimmune Neurology, Mayo Clinic, Rochester, MN, United States; ^3^Department of Laboratory Medicine and Pathology, Mayo Clinic, Rochester, MN, United States; ^4^Division of Neuroscience, IRCCS San Raffaele Scientific Institute, Milan, Italy; ^5^Division of Neurology, Department of Medicine, Siriraj Hospital, Bangkok, Thailand; ^6^Department of Medical, Surgical and Experimental Sciences, University of Sassari, Sassari, Italy

**Keywords:** neuromyelitis optica (NMO), progressive MS, myelin oligodendrocyte glycoprotein (MOG) antibody associated disease, myelopathy, MRI

## Abstract

Inflammatory myelopathies can manifest with a combination of motor, sensory and autonomic dysfunction of variable severity. Depending on the underlying etiology, the episodes of myelitis can recur, often leading to irreversible spinal cord damage and major long-term disability. Three main demyelinating disorders of the central nervous system, namely multiple sclerosis (MS), aquaporin-4-IgG-positive neuromyelitis optica spectrum disorders (AQP4+NMOSD) and myelin oligodendrocyte glycoprotein-IgG associated disease (MOGAD), can induce spinal cord inflammation through different pathogenic mechanisms, resulting in a more or less profound disruption of spinal cord integrity. This ultimately translates into distinctive clinical-MRI features, as well as distinct patterns of disability accrual, with a step-wise worsening of neurological function in MOGAD and AQP4+NMOSD, and progressive disability accrual in MS. Early recognition of the specific etiologies of demyelinating myelitis and initiation of the appropriate treatment is crucial to improve outcome. In this review article we summarize and compare the clinical and imaging features of spinal cord involvement in these three demyelinating disorders, both during the acute phase and over time, and outline the current knowledge on the expected patterns of disability accrual and outcomes. We also discuss the potential implications of these observations for patient management and counseling.

## Introduction

Inflammatory myelopathies or myelitis are a common manifestation of several inflammatory demyelinating disorders of the central nervous system (CNS) and often represent major contributors to neurological disability (1). It is now recognized that the distinct pathophysiological mechanisms underlying these disorders lead to different patterns of spinal cord damage and accrual of neurological deficits (2–4). In multiple sclerosis (MS), the most common demyelinating CNS disorder, recovery from acute myelitis attacks is typically complete or nearly complete, but patients may develop secondary progressive disability over time often in the form of a progressive myelopathy and likely as a long-term consequence of spinal cord damage. Conversely, in neuromyelitis optica spectrum disorder associated with antibodies to the aquaporin-4 water channel (AQP4+NMOSD), and in myelin oligodendrocyte glycoprotein-IgG associated disease (MOGAD), neurological deficits are mainly the result of incomplete recovery from clinical attacks, with a more favorable outcome generally observed in patients with MOGAD. In addition, there is a proportion of presumed demyelinating myelitis for which a clear etiology cannot be identified, and pathophysiology and outcomes remain less clear (5, 6). These include idiopathic inflammatory myelopathies that can occur in isolation (variably referred to as idiopathic transverse myelitis, or idiopathic myelitis) or in the context of etiologically less characterized CNS inflammatory syndromes (e.g., seronegative acute disseminated encephalomyelitis). Lastly, there is a number of less common causes of spinal cord inflammation where demyelination does not represent the main pathophysiologic event (i.e., non-demyelinating myelitis). These include spinal cord sarcoidosis (7, 8), paraneoplastic myelopathies (9), myelitis associated with glial fibrillary acidic protein (GFAP)-IgG (10), and myelitis associated to systemic immune-mediated disorders (e.g., Behcet, systemic lupus erythematosus) (11, 12). A detailed description of the characteristics of non-demyelinating inflammatory myelopathies is beyond the scope of this article, but it has extensively been reviewed elsewhere (1, 13, 14). In this review we will summarize the current knowledge of the myelitis associated with the three most well-characterized demyelinating CNS disorders, namely MS, AQP4+NMOSD and MOGAD, describe their clinical and imaging features and discuss the short- and long-term outcomes.

## Clinical-MRI manifestations

In patients with suspected demyelinating myelitis, a thorough evaluation of the clinical-MRI characteristics is crucial to orient the diagnostic work-up, reduce the risk of misdiagnosis, and promptly initiate the most appropriate treatment. This is particularly relevant in AQP4+NMOSD and MOGAD, where antibody testing is needed to confirm the diagnosis, but often not readily available. In these cases, a correct assessment of the patient with myelitis based on symptoms severity and MRI findings can guide the type and timing of empiric immunotherapy while waiting for antibody testing results. Tables 1, 2 summarize the main clinical, laboratory and MRI features of patients with AQP4+NMOSD, MS, and MOGAD.

**Table 1:** Clinical and CSF findings in patients with AQP4-IgG-positive neuromyelitis optica spectrum disorders (AQP4+NMOSD), MOG-IgG-associated disease (MOGAD), and multiple sclerosis (MS).

	**AQP4+NMOSD**	**MOGAD**	**MS**
**Demographics**			
Typical age at first myelitis	40	20–40	20–40
Sex (F:M)	9:1	1:1	3:1
Ethnicity	Any; African-American/Afro-Caribbean, and Asian more affected	Any; Unclear Ethnic differences	Any; Caucasian more affected
**Clinical features**			
Antecedent infection/immunization	Rare	Common	Rare
Disease course	Relapsing (>95%); a progressive course is rare	Monophasic (50%) or relapsing (50%); a progressive course is rare	Relapsing (85%) or progressive from onset (15%); relapsing patient may develop secondary progression
Myelitis symptoms*			
Weakness	>70%	>70%	40–50%
Sensory disturbances	>70%	>70%	>90%
Trunk sensory level	Common (typically complete)	Common (typically complete)	Common (typically partial)
Bowel/bladder dysfunction	50–60%	60–80%	40–50%
Erectile dysfunction in men	10–20%	>50%	Rare
EDSS ≥ 7 at myelitis nadir	30%	30%	Rare
Recovery from myelitis attacks	Often incomplete	Generally good	Generally good
Need for gait aid long term	40%	5–10%	10–15%
**Extra spine manifestations**			
Optic neuritis	+ + +	+ + +	+ +
Area postrema syndrome	+ +	+ /–	Rare
Encephalopathy	+ /-	+ +	Rare
Focal brain symptoms	+ /-	+	+ +
**CSF findings acutely**			
Oligoclonal bands	<15% (transient)	<15% (transient)	>85% (persistent)
White cell count > 5/μl	>50%	>50%	20–30%
White cell count > 50/μl	20–50%	20–50%	Rare

CSF, cerebrospinal fluid; EDSS, expanded disability status scale-score.

* The frequency of symptoms is estimated considering the first myelitis episode to avoid biases due to spinal cord damage accumulation from multiple myelitis attacks.

**Table 2:** Spinal cord MRI abnormalities in patients with AQP4-IgG-positive neuromyelitis optica spectrum disorders (AQP4+NMOSD), MOG-IgG-associated disease (MOGAD), and multiple sclerosis (MS).

	**AQP4+NMOSD**	**MOGAD**	**MS**
**Spinal cord MRI**			
Longitudinally extensive sagittal T2 lesions*	>85%	80%	Rare
≥2 cord lesions	Uncommon	40–50%	50–60%
Axial T2 abnormalities	Holo-cord	Holo-cord	Peripheral lesions, typically dorsal or lateral
		H-sign	
Acute T1 hypointensity	Common	Rare	Rare
Gadolinium enhancement acutely	>90%; patchy or ring-like	50%; generally nonspecific	>80%; nodular or ring-like
Conus medullaris involvement	10–15%	30–40%	5–30%
Acute MRI initially normal	Rare	~10%	Rare
**Follow-up MRI**			
T2-lesion resolution	Rare	50–80%	Rare
New asymptomatic T2 lesions	Rare	Rare	Common**
Residual T1 hypointensity	Uncommon	Rare	Rare
Persistent gadolinium enhancemet > 6 month	Rare	Rare	Rare
Atrophic evolution of lesions	Common, diffuse	Rare	Common, focal lesion atrophy

* ≥3 contiguous vertebral body segments.

** Although the occurrence of new asymptomatic lesions is common in MS, its frequency is strongly reduced in patients receiving highly effective disease modifying therapies.

### Clinical features of acute myelitis attacks

Various combination of subacute motor, sensory and autonomic dysfunction are the clinical correlates of spinal cord inflammatory lesions. Myelitis symptoms in demyelinating syndromes typically develop subacutely, reaching the nadir between days to few weeks (15), are followed by a phase of plateau/stability, and a subsequent variable extent of recovery. Worsening of neurological deficits beyond three/four weeks is uncommon, and should prompt consideration of different etiologies, including sarcoidosis, malignancy, metabolic or paraneoplastic myelopathies (13, 16).

The typical manifestation of spinal cord involvement in MS is a partial transverse myelitis. Due to frequent involvement of the posterior columns and the cervical spinal segments, predominant sensory symptoms are common, most frequently numbness or paresthesia's, while complete sensory loss is rare. A sensory level can be detected that can be unilateral (e.g., hemi-chest or hemi-abdomen) or bilateral, depending on the location of MS myelitis lesions. The characteristic Lhermitte's sign (an electrical sensation that radiates down the spine or into the extremities triggered by neck flexion) can be observed both acutely and after the acute phase of the myelitis, likely due to demyelinating lesions along the dorsal columns. Motor symptoms, when present, are more commonly unilateral (e.g., lower limb monoparesis with thoracic lesions, upper limb or hemiparesis with cervical lesions), sometimes associated with sensory symptoms to configure a more or less complete syndrome of hemicord injury (Brown-Séquard syndrome). The McArdle's sign (rapidly reversible weakness induced by neck flexion) can also be observed in patients with MS (17). Autonomic deficits are also common, often in the form of bowel/bladder dysfunction with urge, incontinence, constipation/retention, and/or erectile dysfunction in men. The severity of these symptoms in MS is typically mild to moderate, and wheelchair dependence or requirement for bladder catheterization are rare following a single attack, even at symptoms nadir (18, 19). Not infrequently, spinal cord lesions are detected in patients with MS undergoing MRI evaluation for symptoms in other CNS locations, suggesting that spinal cord lesions can be either asymptomatic or pauci-symptomatic at the time of their first development (20, 21). The episodes of myelitis in MS can be clinically isolated or occur in conjunction with symptoms attributable to other CNS locations or asymptomatic brain lesions on brain MRI. In patients presenting with clinically isolated spinal cord involvement suggestive of MS (also see “Spine MRI features” below), a regular brain MRI follow-up is recommended as the occurrence of new asymptomatic brain lesions over time allows fulfillment of the MS diagnostic criteria. The probability of subsequent “conversion” to MS is higher in patients with CSF-restricted oligoclonal bands (OCB) and/or multifocal spinal cord involvement (5, 22, 23). It remains unclear whether those patients manifesting with persistent isolated spinal cord involvement, and thus never fulfilling MS diagnostic criteria, experience a limited form of the disease, or represent instead expression of an entirely distinct disease entity (5, 23, 24).

In contrast to MS, the massive inflammation associated with attacks in AQP4+NMOSD is responsible for severe, typically complete spinal cord impairment (complete transverse myelitis). Motor deficits are frequently bilateral, manifesting as severe para- or quadriparesis, and are commonly associated with sensory loss below the level of the spinal cord lesion (complete trunk sensory level), sphincter and erectile dysfunction. Back pain is frequently reported at presentation, typically at the level of the lesion (25, 26). Pruritus has been reported in up to 27% of patients with myelitis, and can be the presenting symptom during an attack (27). Paroxysmal tonic spasms (recurrent episodes of painful contractions of the trunk or extremities, lasting seconds to minutes that can be triggered by movement or hyperventilation) are described in 25–40% of patients and are characteristic in AQP4+NMOSD, usually developing weeks after the acute myelitis (25, 28). The myelitis episodes are frequently debilitating at nadir (EDSS ≥ 3 in over 50% of cases, EDSS ≥ 7 in over 30%) (26, 29, 30), can manifest with respiratory failure requiring ventilatory support in up to 2% of patients, due to severe cervical myelitis, and be lethal in a minority of cases (31, 32). In up to 4% of cases, the myelitis episodes are associated with concomitant optic neuritis (ON, often bilateral), and around 12% of cases present with simultaneous brainstem manifestations, typically in the form of intractable nausea, vomiting or hiccups lasting more than 48 hours (i.e., area postrema syndrome) (26, 33, 34), although symptomatic brain lesions are also possible (35, 36). A recent retrospective review including over 600 subjects with AQP4+NMOSDs from Denmark, Germany, South Korea, United Kingdom, United States, and Thailand, identified race-related differences in attacks severity, with nadir EDSS score ≥6.0 (unilateral assistance necessary for ambulation) at onset occurring more frequently in Afro-American/Afro-European subjects (58%) as compared to Asian (46%) and Caucasians (38%) (*p* = 0.005) (33). This data need to be confirmed and can be related to socio-economic differences, different access to treatment and timing of treatment initiation.

Myelitis attacks in MOGAD are also frequently severe at nadir, approaching the disability observed in AQP4+NMOSD attacks (EDSS ≥ 7 in over 30%) (19, 26, 29). Motor and sensory symptoms are usually bilateral, and accompanied by bladder dysfunction (usually urinary retention requiring catheterization), neurogenic bowel, and/or erectile dysfunction. Characteristic of MOGAD myelitis compared to AQP4+NMOSD is a greater frequency of sphincter dysfunction likely due to a more frequent involvement of the lower portions of the spinal cord (26). The requirement for mechanical ventilation has been reported in 3% of cases, but in contrast to AQP4+NMOSD where it was secondary to severe cervical involvement, respiratory failure in subjects with MOGAD was more commonly due to profound encephalopathy and seizures (typically in the context of acute disseminated encephalomyelitis) (31). Tonic spasms and severe neuropathic pain associated with myelitis are less common compared to AQP4+NMOSD (28). Isolated MOGAD myelitis is the second most frequent phenotype in adults, accounting for around 30% of presentations, while it accounts for 11% of cases in children. An additional 7% of adult and 6% of pediatric cases present with myelitis in association with concomitant ON, and 6% of adults and 18% of children report myelitis symptoms in the context of ADEM or multifocal CNS involvement without encephalopathy, although the frequency of asymptomatic spinal cord lesions in these contexts can be higher (20, 26, 34, 37–41). A prodromal infection or vaccination are common before MOGAD attacks, described in up to 57% of cases, usually consisting in variable combinations of rhinorrhea, sore throat, low-grade fever, malaise, and cough (19). Some patients can present with flaccid are flexia due to involvement of the anterior horns, resembling acute flaccid myelitis (19). Intractable nausea or vomiting can be observed in the context of ADEM, but they are rarely observed in isolation and, in contrast to AQP4+NMOSD, are not usually associated with presence of discrete lesions in the area postrema (42, 43).

### Spine MRI features during acute attacks

**Figure 1:**
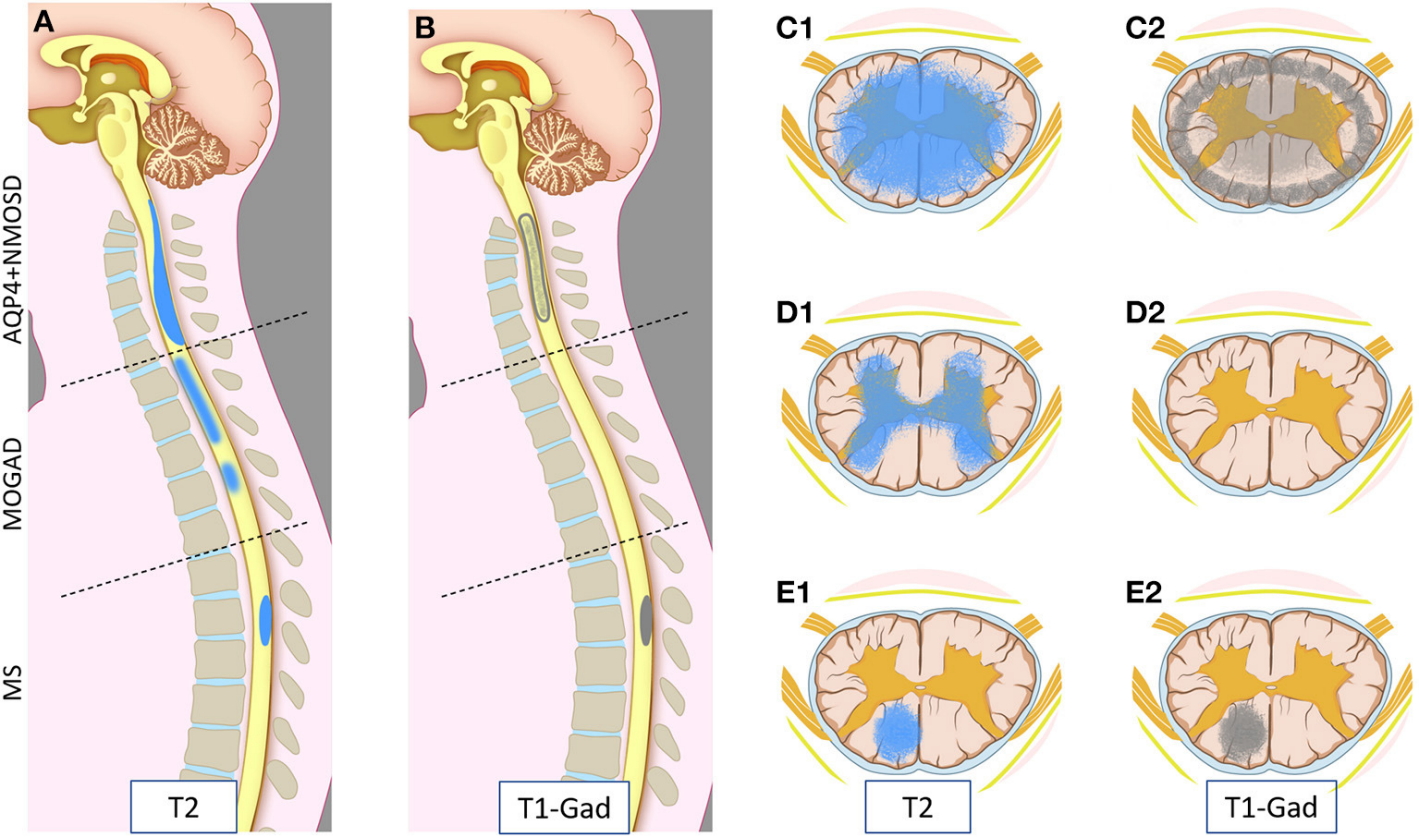
Schematic representation of myelitis lesion location and gadolinium enhancement patterns on spinal cord MRI in AQP4-IgG-positive neuromyelitis optica spectrum disorder (AQP4+NMOSD), MOG-IgG associated-disease (MOGAD), and multiple sclerosis (MS). Sagittal T2 **(A)** and T1 post-gadolinium **(B)** MRI images (left half); and axial T2 **(C1,D1,E1)** and T1 post-gadolinium **(C2,D2,E2)** images (right half) are schematically represented. Patients with AQP4+NMOSD myelitis typically show a single longitudinally extensive lesion in the cervical spinal cord [**(A)** upper part] with extensive parenchymal involvement axially **(C1)** on T2 weighted sequences. These lesions typically show gadolinium enhancement during attacks, often at the periphery of the T2 lesion with a ring-like pattern axially **(C2)** or “elongated ring” when appreciated on sagittal images [**(B)** upper part]. In MOGAD, myelitis lesions can affect any spinal cord level with similar frequency. Short and longer T2-lesions can coexist (not shown) [**(A)** central part]; while acute lesion enhancement is absent in ~50% of cases [**(B)** central part, **(D2)**] and when present is often nonspecific. On axial images, T2 abnormalities often predominantly involve the central gray matter (H-sign) **(D1)**. Lastly, MS myelitis lesions are typically short on sagittal T2 images [**(A)** bottom part], often showing ring or nodular/ovoid enhancement during acute attacks [**(B)** bottom part, **(E2)**]. On axial images, T2 lesions typically affect the periphery of the spinal cord along the dorsal or lateral columns that clinically results in a “partial transverse myelitis” **(E1)**. All three diseases can involve the entire spinal cord, and different lesion locations in this figure are intended for graphic purposes.

**Figure 2:**
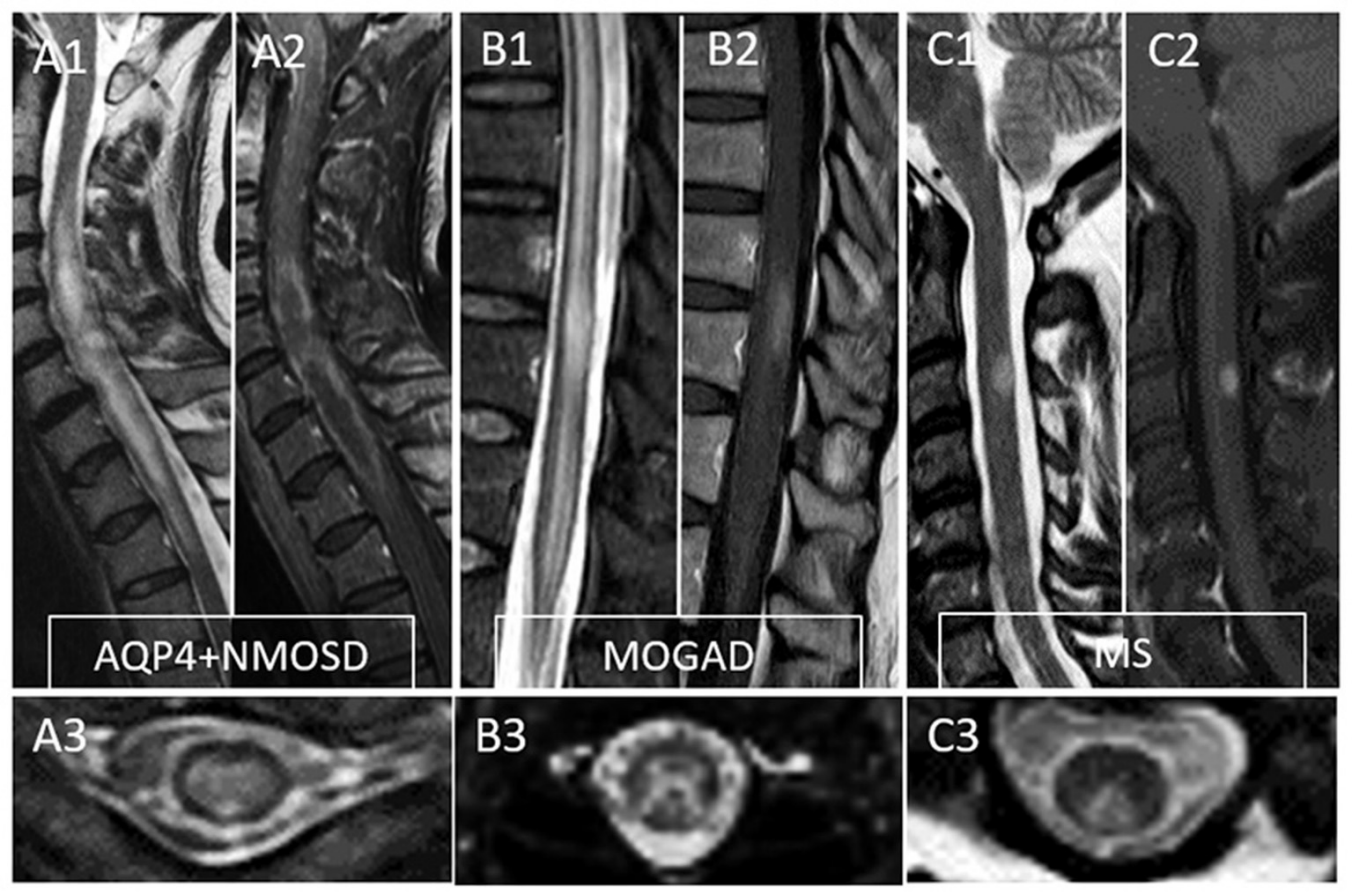
Typical MRI findings in patients with myelitis associated with AQP4-IgG-positive neuromyelitis optica spectrum disorder (AQP4+NMOSD), MOG-IgG associated disease (MOGAD), and multiple sclerosis (MS). A typical longitudinally extensive T2-lesion affecting the cervical spinal cord in a patient with AQP4+NMOSD is evident on sagittal images **(A1)**, with peripheral enhancement after gadolinium administration—“elongated ring” enhancement **(A2)**. Axially, the T2 lesion involves the majority of the spinal cord **(A3)**. In patients with MOGAD myelitis, involvement of the conus medullaris by the longitudinally extensive T2 lesions **(B1)** is significantly more common compared to NMOSD, with accompanying patchy or nonspecific enhancement in about half of cases **(B2)**. On axial T2 images, a predominant involvement of the central gray matter is common forming a H-sign **(B3)**. Lastly, MS myelitis T2 lesions are typically short on sagittal images **(C1)** and peripherally located along the dorsal **(C3)** or lateral columns axially. After gadolinium administration, lesion enhancement can be nodular **(C2)** or ring-like.

MRI provides essential clues for the differential diagnosis of inflammatory myelopathies, and by virtue of being readily available before the results of antibody testing, it is key in guiding the first treatment decisions. Figure 1 schematically shows the typical location and gadolinium enhancement patterns of different demyelinating lesions on spinal cord MRI, and representative MRI images are shown in Figure 2.

The typical imaging features of MS-associated myelitis are short T2-lesions defined as spanning <3 vertebral segments (usually extending over <2 vertebral segments), single or multiple, with preferential involvement of the cervical segment (although any level can potentially be affected) (44). Longitudinally extensive lesions (spanning ≥3 contiguous vertebral body segments) accompanying the myelitis (also known as longitudinally extensive transverse myelitis or LETM) are extremely rare and should prompt considering alternative etiologies. Of note, the confluence of a large number of short contiguous lesions can sometimes resemble a LETM on sagittal images, but careful assessment of lesions on axial views allows to distinguish the individual discrete short lesions (20, 45); also hazy T2-hyperintensity can be noted, particularly in chronic MS and individual discrete T2-lesions may be difficult to identify. While the occurrence of LETM has previously been reported in up to 17% of children with MS, this proportion did not exceed 5% in a pediatric MS cohort where MOGAD was carefully excluded (20). On axial views, lesions are typically wedge shaped, located at the periphery of the spinal cord, with predilection for the posterior and lateral columns (44). Although most MS lesions involve a combination of spinal cord white and gray matter, the presence of lesions restricted to the spinal cord white matter is highly suggestive for MS as compared to other demyelinating etiologies (20). Lesions hyperintense on T2-weighted sequences are typically isointense on conventional T1-weighted images (46), but T1-hypointensity can be observed at high field strength (47) or with specific sequences (e.g., 3D-Phase Sensitive Inversion Recovery, 3D-PSIR) (48). Acute spinal cord lesions typically enhance after administration of gadolinium, although less frequently than brain lesions, and can demonstrate nodular or ring enhancement within the spinal cord (49).

Several elements differentiate the typical imaging features of AQP4+NMOSD from MS. A single, longitudinally extensive lesion (i.e., spanning over three or more vertebral segments) is observed in over 80% of cases (50), but lesions may appear short in ~14% of cases, particularly when imaged during the phase of development or resolution (33, 51–53). During myelitis attacks, T1-hypointensity can be observed even with conventional sequences, in contrast to the typical T1 isointensity observed in MS or MOGAD spinal cord lesions (54). Lesions in AQP4+NMOSD predominantly appear centrally located on axial views. Cervical lesions may sometimes extend rostrally to involve the medulla and, although this can also be seen with longitudinally-extensive T2-lesions of other etiologies, the coexistence of spinal cord dysfunction and intractable nausea/vomiting with from area postrema involvement is highly suggestive for AQP4+NMOSD (55). Tumefactive enlargement of the spinal cord is relatively frequent during the acute phase (50), and can be followed by cavitation and necrosis in cases of severe disease. Gadolinium enhancement is present in almost all myelitis lesions acutely, with variable patterns but typically localizing at lesion margin, with ring or elongated ring enhancement pattern observed in about 30% of cases (46). A characteristic imaging finding is the presence of bright spotty lesions, defined as areas of higher T2-yperintensity within the spinal cord lesion that are comparable to the signal intensity of the cerebrospinal fluid (CSF) (56, 57). This finding has shown specificity for AQP4+NMOSD of 94% [95% CI (85.6–97.7)] vs. a range of inflammatory and non-inflammatory myelopathies, and a sensitivity of 40% [95% CI (19.8–64.3)] (58).

The imaging features of myelitis in MOGAD have been well delineated during the last few years. Similarly to AQP4+NMOSD, lesions are typically longitudinally extensive, but short lesions are detected in over one fourth of subjects, often in combination with LETM (20, 26). Lesions can involve most of the spinal cord length, spanning over a median of 7 vertebral segments in adults and 10 segments in children in some studies (20, 26). In up to 10% of MOGAD cases presenting with clinical features of myelitis, the initial spinal cord MRI does not demonstrate parenchymal T2-signal abnormalities or enhancement (59). In these patients with initially negative MRI, spinal cord abnormalities may appear on subsequent scans obtained after a few days. The most characteristic imaging feature in MOGAD myelitis is the prominent involvement of the ventral spinal cord on sagittal images (ventral sagittal line), and of the spinal cord gray matter axially, which configures the so called H-sign (19). The H-sign has been reported in 30–60% of MOGAD myelitis, and helps in distinguishing MOGAD from AQP4+NMOSD, where it is observed in 33% of cases, and particularly MS, where its occurrence is exceptionally rare (19, 20). Gray matter involvement can be associated with surrounding fainter white matter hyperintensity, particularly in the central portion of the lesion, while the gray matter hyperintensity is more prominent at the upper and lower extremities (20). Although the upper spinal cord segments are frequently involved, a more frequent involvement of the conus can help distinguishing MOGAD myelitis from other demyelinating etiologies (19, 20, 26). Presence of acute lesional enhancement is seen in ~50% of cases (19, 26), while leptomeningeal enhancement has been reported in up to 70% of children (20), sometimes extending to the cauda equina nerve roots (20, 60). The frequency of leptomeningeal enhancement in adults with MOGAD-associated myelitis is less clear (60, 61).

### CSF findings

In addition to MRI acquisition, CSF analysis often provides important clues to guide the differential diagnosis of myelitis and is recommended in the diagnostic workup of subacute myelopathies. Although not strictly necessary for the diagnosis of a specific demyelinating disease, it often provides useful information for an early identification of a specific syndrome, and for excluding other non-inflammatory and infectious etiologies (62).

The presence of intrathecal IgG synthesis, in the form of increased IgG index or CSF-restricted OCB is a characteristic feature of MS, being observed in over ~85% of cases (63). Although the frequency of CSF OCB was previously considered to be lower in pediatric than in adult-onset MS (64), studies conducted on children with MS carefully assessed for the absence of MOG-IgG revealed a frequency similar to the one observed in adult cohorts (65). CSF pleocytosis (i.e., cell count >5/mm^3^) with lymphocytic predominance is typically reported in about 50% of MS cases, while cell counts above 50/mm^3^ are exceptionally rare and should prompt consideration for alternative diagnoses.

Conversely, CSF OCB are observed in a minority of subjects with AQP4+NMOSD and MOGAD (10–20%), and can disappear during the remission phase (19, 66). CSF pleocytosis is observed in 14–79% of patients with AQP4+NMOSD, exceeding 50 cells/mm^3^ in 13–35% of cases. It usually demonstrated monocytic and lymphocytic predominance, but neutrophils and eosinophils can also be detected in variable proportions. Patients with MOGAD also demonstrate pleocytosis in around 50–75% of cases acutely, with white blood cells count above 50 cells/mm^3^ in 19%, and ≥100 cells/ mm^3^ in 12% of cases (66). While the predominant cell types are lymphocytes and monocytes, neutrophils can be detected in up to 40% of cases, and eosinophils are rare (<3% of cases) (66). In both AQP4+NMOSD and MOGAD, CSF findings are related to the clinical phenotype at the time of the sample collection, with less prominent abnormalities observed in subjects with isolated ON attacks (66, 67).

## Outcomes

After the acute phase of the myelitis, both clinical and MRI evolution vary substantially between AQP4+NMOSD, MOGAD and MS. The first months following the myelitis are generally characterized by some degree of improvement, influenced by the specific disease, features of the myelitis attack and the patient's demographic profile, while the long-term outcome is generally determined by the occurrence and frequency of clinical relapses and, in patients with MS, by the development of gradual worsening of disability, often in the form of progressive spinal cord dysfunction.

### Recovery from acute attacks

The clinical recovery from a myelitis is variable based on the initial severity and underlying etiology, and may continue for over 2 years after the attack. The most noticeable improvements, however, occur within the first months, and the chances for further recovery substantially decrease if no improvements are made within the first 3–6 months (68). Prompt initiation of an adequate treatment aims at speeding up recovery and minimizing the residual deficits.

MS relapses usually cause transient neurologic dysfunction, with 38–66% of cases showing complete recovery (69–72). The degree of recovery from each attack is likely multifactorial, resulting from a combination of subject-specific and relapse-related factors (73). Greater severity of symptoms at nadir, simultaneous presence of symptoms attributable to multiple CNS locations and older age have been associated with worse outcome (69, 71, 74–76). In particular, the recovery from MS attacks is usually greater in children than in adults (77), despite pediatric patients presenting on average with more severe symptoms at nadir. In a prospective study considering over 200 attacks in 72 subjects with clinically isolated syndrome or MS, the probability of residual deficits varied according to the presence of specific symptoms, being 45% for sphincter dysfunction, 34% for sensitive and 24% for motor symptoms (71). The precise impact of acute treatment on the degree of clinical recovery from MS-associated myelitis is currently unclear. One study assessed the effects of immediate initiation of interferon beta-1a therapy soon after an initial MS attack (consisting in myelitis in 22% of participants), and observed that early treatment initiation was associated with greater likelihood of preserving long term ambulatory ability in those subjects who experienced initial poor recovery (72).

Similarly to MS, the degree of recovery from AQP4+NMOSD attacks is influenced by age and attack severity, with better recovery observed in younger individuals with lower deficits at nadir (26, 78–80). In a retrospective study on 182 subjects with AQP4+NMOSD, the length of the longest spinal cord lesion measured on MRI was found to be a predictor of more severe residual disability (81), while another study showed that CSF levels of glial fibrillary acidic protein during acute attacks correlated with the length of spinal cord lesions and with the EDSS at 6 months from the index attack (82). As discussed above, ethnicity might also influence clinical outcome, with Caucasians being reported at greater risk of severe disability than Japanese individuals in one study (83), and African-Americans being found at increased risk of respiratory failure and death in north American studies (where inequity in access to and quality of health care might be a contributing factor) (83, 84). How the differences in the severity of the acute attack affect long-term outcome is less clear, and likely even more influenced by different treatment paradigms. Subjects with AQP4+NMOSD are overall more likely to experience partial, often poor recovery from each myelitis attack, and a myelitis presentation is associated with greater likelihood of reaching severe motor disability compared to isolated ON (85). The presenting attack is responsible for reaching EDSS ≥ 6 and EDSS ≥ 8 in about 25 and 20% of all subjects ever reaching these disability milestones, respectively (83). The type and timing of acute treatment for AQP4+NMOSD attacks plays a critical role not only in accelerating recovery, but also in increasing the remission rate. In a retrospective study on 871 NMOSD attacks, complete recovery was obtained in only 19% of patients after first line treatment, but in an increasing proportion of cases following additional treatment courses (85). In 84% of cases, the first line treatment consisted in high-dose intravenous steroids and in 8% of cases in plasma exchange, while this latter treatment was increasingly used as second (34%) and subsequent line treatments (>50%). Evidence exists on the importance of prompt initiation of plasma exchange for treatment of acute NMOSD attacks, as the probability of achieving complete recovery decreases with the delay in plasma exchange initiation, going from a 50% improvement at day 0–1 to ~5% after 20 days (86). In clinical practice, plasma exchange should be strongly considered in patients with moderate-severe disability, especially when they do not show prompt improvement and consistent improvement after the first 1–2 days of treatment with intravenous corticosteroids.

Subjects with MOGAD have typically good recovery from myelitis attacks, particularly of motor symptoms, with only 6% of patients requiring walking aid at last follow up (19). Age at the time of attack seems to be a predictor of the post-acute outcome, with faster and greater recovery observed in children compared to adults (38). In a study comparing outcomes of myelitis attacks in AQP4+NMOSD and MOGAD, the median EDSS at myelitis recovery was 3.0 (range 1.0–8.0) for AQP4+NMOSD and 1.8 (range 1–8.0) for MOGAD, with only 7% of patients having an EDSS ≥ 6 at recovery in the MOGAD group vs. 44% of the AQP4+NMOSD (26). Despite good motor recovery, MOGAD myelitis is associated with a relatively high proportion of persistent sphincter dysfunction (44–59%, in the form of urinary urgency, hesitancy, incontinence, frequent urinary tract infections, fecal incontinence or constipation), and around 25% of patients require long term catheterization (19, 26, 87). The need for catheterization was associated with the presence of conus lesions, but not with the severity of the myelitis episode or the EDSS score at last follow-up (26). Although the overall frequency of sphincter dysfunction did not differ between subjects with MOGAD and AQP4+NMOSD, its presence as a residual deficit in isolation was more common in subjects with MOGAD. Erectile dysfunction is also common among male patients with MOGAD (46%), and is significantly more common than in those with AQP4+NMOSD (26).

### Disease course

Important differences have recently been highlighted in the natural history of these three demyelinating disorders, which ultimately influence the long-term prognosis and contribution of individual attacks to it.

The disease course in MS is initially characterized by discrete clinical attacks followed by complete or nearly complete recovery, interspersed with periods of disease stability. This form of disease, referred to as “relapsing–remitting,” accounts for about 85% of all MS cases. Over time, relapses decrease in frequency and after an average of 15 years from onset most patients in historical cohorts enter the so called “secondary progressive” phase of the disease, with gradual deterioration of their neurological function independent from clinical relapses (88). In 10–15% of adult MS cases, the disease can manifest with progressive clinical deterioration from onset; this “primary progressive” phenotype is exceedingly rare in children (89). While the exact mechanisms underlying the development of progressive disability (either primary or secondary progression) in MS remain unclear, it typically manifests as progressive, asymmetric motor impairment anatomically localizing to the spinal cord (90, 91). This association can be best appreciated in rare patients with a single CNS demyelinating lesion in the spinal cord (also known as “progressive solitary sclerosis”) and worsening motor impairment from onset that anatomically localize to that lesion (92, 93). Although these patients with progressive solitary sclerosis do not meet MS diagnostic criteria (a minimum of two spinal cord lesions is required for a primary progressive MS diagnosis) (94), a similar anatomical association is seen in MS patients with highly restricted CNS lesion burden (<5 lesions) (95), or rare MS patients with exclusively unilateral motor impairment (96). In all these patients, at least one demyelinating lesion can often be identified along the spinal cord lateral columns which is anatomically explanatory for the progressive motor impairment. These lesions, also referred to as “critical corticospinal tract lesions,” are typically large and accompanied by focal atrophy (also see the “Follow-up MRI findings” section) (92, 93, 95–100). Notably, a primary progressive disease course may also be observed in patients initially diagnosed with radiologically isolated syndrome (MRI detection of typical MS lesion in the absence of accompanying clinical manifestations), and all of these patients have MRI evidence of spinal cord involvement before symptoms onset (101). This finding suggests that spinal cord lesions in MS may sometimes develop asymptomatically and subsequently induce structural spinal cord changes (e.g., chronic inflammation, focal lesional atrophy) eventually resulting in progressive myelopathy.

Progressive neurodegeneration is not considered to be a major component of AQP4+NMOSD pathophysiology (2), while the accrual of neurological deficits is closely linked to the occurrence of severe relapses (stepwise disability accrual). Over 95% of subjects with AQP4+NMOSD experience a relapsing course. The risk of a first relapse has been estimated around 60% within the first year from a myelitis presentation (102), with lower risk for relapses associated with increasing age and onset with brainstem symptoms (103, 104). Although cases of monophasic disease have been reported, as well as others with disease quiescence for more than a decade (53, 105, 106), these are extremely rare and all subjects diagnosed with AQP4+NMOSD should be considered at high risk for recurrence, and should be offered a preventive therapy protracted long-term, as withdrawal of treatment has been found to increase the risk of relapse even after long periods of remission (107, 108).

Data on the long-term clinical course of MOGAD are still limited. However, evidence from large prospective cohorts demonstrate a monophasic course in around 50% of cases, more commonly in children than in adults (38, 109). Current figures estimate that about 40% of adults and 30% of children would have a second clinical attack within 5 years from first presentation (110), and the risk for relapses in untreated patients is considered higher in the first 4 years post-onset compared to the subsequent 4 years [HR 0.21 (95% CI, 0.06–0.77)] (111). Among relapsing cases, the inter-attack interval can be highly variable, ranging from few months to many years (111–113). In the setting of such a variable disease course, definitive predictors of clinical relapses have not been identified. Apart from age at disease onset, evolution of serological status has shown a moderate association with disease course, with subjects becoming seronegative for MOG-IgG being on average at lower risk for further relapses (109, 113). In a cohort study on 276 subjects with MOGAD, the presence of myelitis at onset, alone or in combination with other presentations, was associated with decreased risk of relapse compared to other non-myelitis phenotypes (HR, 0.41; 95% CI, 0.20–0.88; *P* = 0.01) (114). The most common phenotype of MOGAD relapses is ON, regardless of the initial clinical presentation, and recurrent episodes of myelitis as the sole clinical phenotype of MOGAD are uncommon (115, 116). Although evidence is still limited, a relapse-independent disease progression is considered extremely rare in MOGAD, and a gradually progressive disease course should prompt careful consideration of alternative diagnoses (117). This is extremely relevant in MOGAD where the risk of misdiagnosis is higher compared to AQP4+NMOSD due to the possibility of false MOG-IgG positivity (which is almost negligible with AQP4-IgG) (55, 118, 119).

### Follow-up MRI findings

Although in most cases the spinal cord MRI findings in the acute phase already provides valuable clues for diagnosis, the evolution of imaging features over follow-up exams is often useful to clarify or confirm the diagnostic suspect.

**Figure 3:**
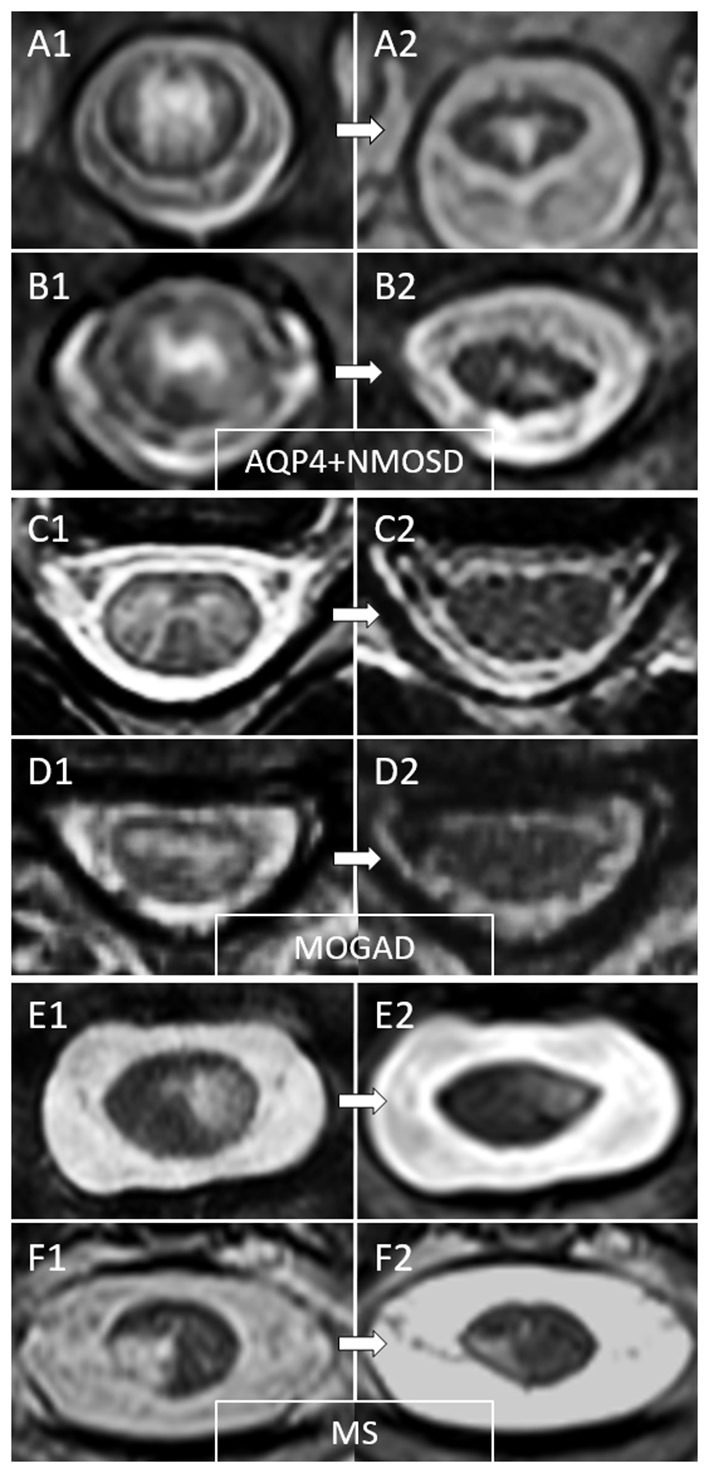
Examples of temporal evolution of T2-lesions axially on spinal cord MRI in AQP4-IgG-positive neuromyelitis optica spectrum disorder (AQP4+NMOSD), MOG-IgG-associated disease (MOGAD), and multiple sclerosis (MS). Acute myelitis lesions in AQP4+NMOSD typically involve extensively the spinal cord parenchyma **(A1,B1)**. After the acute phase of the myelitis **(A2,B2)**, a residual T2 scarring is common, often accompanied by spinal cord atrophy at lesion level (a clear reduction in spinal cord diameter can be noted at follow-up MRI). In MOGAD myelitis lesions, the T2 abnormalities observed acutely **(C1,D1)** tend to resolve completely at follow-up MRI in the majority of cases **(C2,D2)**. On the contrary, MS T2-lesions tend to persist over time with only minimal reduction in size from the acute phase **(E1,F1)**, sometimes resulting in focal lesion atrophy at follow-up **(E2,F2)**. These focally atrophic lesions are more common in patients with progressive forms of MS and strongly associate with progressive motor impairment when located along the spinal cord lateral columns.

Following a myelitis attack, spinal cord lesions typically evolve by reducing in size over subsequent MRI's and may resolve completely to undetectable, or be accompanied by development of spinal cord atrophy at lesion level. Examples of MRI lesion evolution over time on axial images are shown in Figure 3.

MOGAD lesions demonstrate the greatest reduction in T2-lesion area, followed by lesions due to AQP4+NMOSD, with smaller changes observed in MS lesions size over time (120). Complete resolution of T2 hyperintense spinal cord lesions is uncommon in both MS and AQP4+NMOSD, while it is observed in 62–79% of MOGAD myelitis lesions (20, 26, 120). The median time from the first spinal cord lesion detection to first normal follow-up MRI has been reported to be around 9 months in one study, although this data was limited by the lack of regular and close follow-up MRI exams and complete lesions resolution can be observed as early as 12 days from the first scan (20, 120). No clinical or paraclinical features have been identified as significant predictors of lesion resolution (120). Due to the changes in lesion volume in AQP4+NMOSD myelitis, a single LETM can evolve into multiple shorter lesions in subsequent exams, which can mimic MS (121). Residual T1 hypointensities in the spinal cord are relatively rare across different demyelinating diseases, and in a recent study were observed in 3/34 (9%) of AQP4+NMOSD myelitis, 1/29 (3%) of MS cases, and in none of 28 MOGAD cases (120).

The development of new asymptomatic spinal cord lesions on serial MRI is observed in about 25% of MS patients (122), but is highly dependent on treatments utilized and will likely be encountered much less commonly in the era of high-efficacy disease modifying treatments in MS. By contrast, these were observed in only 1/116 (0.9%) AQP4+NMOSD and 0/81 (0%) MOGAD cases in a recent retrospective longitudinal study (123). In the same study, a slightly higher frequency of asymptomatic spinal cord lesions were observed on MRI scans acquired in concomitance with non-myelitis attacks (3/331, 0.9% in AQP4+NMOSD and 11/151, 7.3% in MOGAD) (123).

Variable degree of spinal cord atrophy can be detected on clinical images in subjects with MS, but spinal atrophy is often a prominent feature on spine MRI exams acquired during remission in subjects with AQP4+NMOSD and history of myelitis, particularly at the level of spinal cord lesions (124–126). In such patients, atrophy can be sometimes associated with frank myelomalacia, which can provide a valuable hint for diagnosis (112). On the contrary, overt spinal cord atrophy is uncommon in MOGAD, although it can be detected in the gray matter corresponding to the level of acute lesions (126). In patients with MS, the development of focal spinal cord lesion atrophy over time is characteristic, often with a strong anatomical association with progressive motor impairment (“Critical corticospinal tract lesions”; also see the “Disease course” section) (96, 97).

Overall, the presence of spinal cord atrophy is better appreciated through the use of quantitative MRI. Studies assessing quantitative MRI metrics in patients with AQP4+NMOSD found that these patients have significant spinal cord atrophy compared to controls (124–126), but also MS and MOGAD patients (125, 126). Atrophy usually localized to areas involved by spinal cord lesions and correlated with the number of myelitis episodes (124–126). Spinal cord areas not previously affected by lesions and AQP4+NMOSD patients without history of myelitis showed comparable metrics to healthy controls (124, 126). Subjects with MOGAD showed a reduction in spinal cord gray matter volume exclusively in areas affected by previous attacks, which was greater in subjects with relapsing compared with monophasic course, even if relapses occurred elsewhere in the CNS. Finally, subjects with MS showed reduced cervical cross-sectional area and gray matter volume compared to controls, without significant association with the presence of lesions, in keeping with the diffuse neurodegenerative changes that characterize this disease (126).

### Long term prognosis

**Figure 4:**
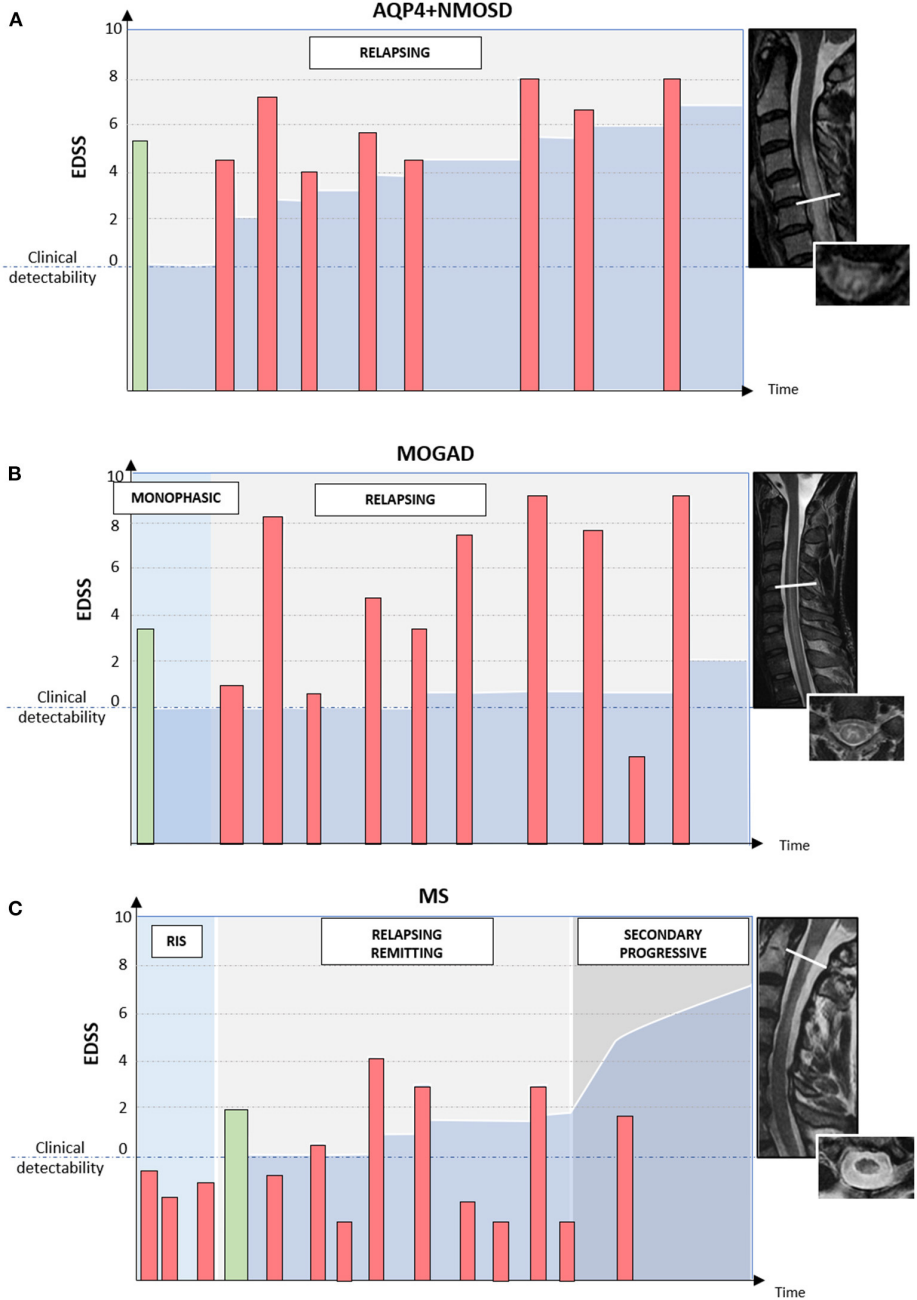
Different disease courses and patterns of disability accrual in AQP4-IgG-positive neuromyelitis optica spectrum disorder (AQP4+NMOSD), MOG-IgG-associated disease (MOGAD), and multiple sclerosis (MS). Red bars = disease relapses; green bars = first clinical attack; darker blue area in the bottom half of each diagram = accumulated disability. In patients with AQP4+NMOSD **(A)** disease relapses are almost always clinically manifested with severe disability and often incomplete recovery between attacks. Disability accumulates in a stepwise fashion. In patients with MOGAD **(B)** disease attacks are similarly severe to those observed in NMOSD but post-attack recovery is generally complete or nearly complete. Long-term outcomes in MOGAD are often favorable with less disability accumulation **(B)**. Lastly, in patients with MS **(C)** disease relapses are typically of mild-moderate severity and often do not cross the threshold for clinical detectability. For this reason, the first clinical attack often occurs in patients with MRI evidence of prior asymptomatic lesions. When these lesions are detected incidentally during the diagnostic workup for other disorders (e.g., headache), before the presenting MS attack, a radiologically isolated syndrome (RIS) can be diagnosed. When clinically manifested, MS attacks tend to result in complete or nearly complete clinical recovery in most cases (relapsing-remitting MS); while progressive disability accumulation can be seen in a minority of cases either from disease onset (primary progressive MS) or after an initially relapsing-remitting course (secondary progressive MS). Progressive disability generally occurs in the form of progressive motor impairment in MS, which often coincides with the development of focally atrophic lesions in the spinal cord lateral columns.

The typical disease course and patterns of long-term disability accrual observed in AQP4+NMOSD, MOGAD, and MS are summarized in Figure 4.

The natural history of MS encompasses a progressive accrual of disability, with an estimated median time from onset to EDSS 6 (i.e., requiring walking assistance) of 28 years (127). This estimate, based on a study on over 2,800 MS patients with symptom onset before 1988, depicts a slower rate of progression compared to data from older MS cohorts with onset before 1964 (128). Such difference is likely in part attributable to the increasing availability of disease modifying therapies from the 1990s, but also to greater recognition and diagnosis of milder forms of the disease. Nonetheless, the actual rate of disability accrual is highly variable at the individual level, and it is difficult to compare between studies due to different designs and epochs in which the studies were conducted (e.g., with different treatments available). According to a population-based study, ~17% of MS patients will have low disability (EDSS score ≤ 2) at 10 years from clinical onset (129), while 6–12% of patients would reach EDSS ≥ 6 within 5 years (130, 131). In particular, up to 13% of individuals are expected to experience an extremely benign course, with only few attacks, each followed by complete recovery, absence of cumulative disability and preservation of the ability to work 15 years from first symptoms (132).

The disease course in the first few years post-onset appears to be an important predictor of the long term outcome, with subjects with short interval from onset to first relapse tending to follow a more rapidly progressive course (133), and the vast majority of subjects with minimal disability at 5 years post-onset continuing to be ambulatory at 15 years (129). In general, subjects with initial presentation before the age of 40 and with sensory symptoms have a better prognosis than those presenting at later age and with pyramidal or cerebellar dysfunction (101, 134). The presence of spinal cord symptoms and incomplete recovery from relapses have also been associated with faster disease progression (88). In particular, even the presence of a single lesion critically located in the high cervical cord can predispose to development of progressive disease (92, 95). From an imaging prospective, the presence of asymptomatic spinal cord lesions in subjects with clinically isolated syndromes (i.e., the first clinical episode suggestive for MS not meeting criteria for dissemination in time) is associated with increased risk for higher disability at 5 years (135). Similarly, in subjects with radiologically isolated syndrome, the presence of spinal cord lesions is associated with higher risk of fulfilling MS diagnostic criteria and of experiencing a primary progressive disease course (136). In multiple studies, spinal cord atrophy showed correlation with greater physical disability and disease progression (137–140).

The long-term prognosis of AQP4+NMOSD has substantially improved since the first years after its characterization, largely thanks to a better control of disease activity through timely initiation of immunotherapy and more frequent use of plasma exchange for acute attacks (32, 106). The disability of individuals with AQP4+NMOSD is nonetheless often greater than the one of subjects with MS with comparable disease duration. At 5 years from presentation, around 22% of cases are expected to have reached an EDSS of 6, and about 8% an EDSS of 8 (i.e., restricted to a wheelchair) (106). This proportion is even higher when considering only AQP4+NMOSD subjects with relapsing LETM, as 36% of them would be expected to reach EDSS of 6 by 5 years from onset (105). The risk of severe motor disability is greater in subjects with AQP4+NMOSD presenting with myelitis than in those presenting with ON (106). It is also influenced by the age at onset (26, 103), with the risk of requiring ambulatory aid being reported to increase by 32% for every decade increase in age (106). Although rare, cases of mild disease course have been reported, with around 12–16% of subjects with AQP4+NMOSD having an EDSS ≤ 3 more than 10 years after first symptoms (106).

In contrast, the long-term prognosis for subjects with MOGAD is usually more favorable. Two studies on 29 and 61 subjects followed for over 8 years from disease presentation (median 14 years) reported an EDSS ≤ 2 at last follow-up in over 50% of participants, and an EDSS ≥ 6 in only 12.5% (3, 115). Between 8 and 12% of participants in these studies had a phenotype of isolated transverse myelitis at their last follow-up, while most participants experienced episodes of ON at least once. Subjects with a final phenotype of isolated ON were more likely to have a final EDSS of <3, while history of both ON and myelitis attacks was associated with greater likelihood of final EDSS ≥ 3 (115). Although the median pyramidal and sphincter function system scores were of 0, a remarkable 12.5% of patients required permanent urinary catheterization. The overall clinical outcome might be more favorable in children than adults. In a study comparing the clinical outcome of 98 children and 268 adults with MOGAD, an EDSS ≥ 3.0 at last follow-up (range 0.5–33 years from presentation) was reached by19% of children with a clinical presentation of myelitis, alone or in combination with ON, and in 42% of adults with analogous presenting phenotype (38). In a cohort of children with MOGAD associated myelitis followed for 5 years after presentation, pyramidal deficits were observed in only 6/22 (27%), and these were in all cases neurological signs without associated disability (functional pyramidal system score of 1) (20).

## Conclusions

The clinical outcome of inflammatory myelopathies varies greatly, and it is influenced by multiple factors. Some determinants of disability are related to the specific disease etiology and to the patient's demographics, while others are potentially modifiable factors, such as the delay in diagnosis, poor treatment or initiation of preventive treatments. Prompt and accurate diagnosis of the specific underlying disease and early initiation of the appropriate acute and relapse preventive treatments are key to limit disability accrual. Thanks to the increasing awareness of the distinctive clinical and imaging features associated with different demyelinating disorders, and to the availability of highly effective therapies, physicians have now access to valuable instruments to significantly alter the final clinical outcome of inflammatory myelopathies.

## Author contributions

GF, EF, and ES: study concept and design, drafting the manuscript and figures, and study supervision. All authors: data collection and interpretation and revised the manuscript for intellectual content. All authors contributed to the article and approved the submitted version.

## Conflict of interest

The authors declare that the research was conducted in the absence of any commercial or financial relationships that could be construed as a potential conflict of interest.

## Publisher's note

All claims expressed in this article are solely those of the authors and do not necessarily represent those of their affiliated organizations, or those of the publisher, the editors and the reviewers. Any product that may be evaluated in this article, or claim that may be made by its manufacturer, is not guaranteed or endorsed by the publisher.

## References

[1] Flanagan EP. Neuromyelitis optica spectrum disorder and other non-multiple sclerosis central nervous system inflammatory diseases. Continuum (Minneap Minn). (2019) 25:815–44. 10.1212/CON.0000000000000742 31162318

[2] Wingerchuk DM Pittock SJ Lucchinetti CF Lennon VA Weinshenker BG A. secondary progressive clinical course is uncommon in neuromyelitis optica. Neurology. (2007) 68:603–5. 10.1212/01.wnl.0000254502.87233.9a 17310032

[3] Lopez-Chiriboga AS Sechi E Buciuc M Chen JJ Pittock SJ Lucchinetti CF. Long-term outcomes in patients with myelin oligodendrocyte glycoprotein immunoglobulin G-associated disorder. JAMA Neurol. (2020) 77:1575–7. 10.1001/jamaneurol.2020.3115 32865549 PMC7489431

[4] Iaffaldano P Lucisano G Patti F Brescia Morra V De Luca G Lugaresi A. Transition to secondary progression in relapsing-onset multiple sclerosis: definitions and risk factors. Mult Scler. (2021) 27:430–8. 10.1177/1352458520974366 33210986

[5] Sechi E Shosha E Williams JP Pittock SJ Weinshenker BG Keegan BM. Aquaporin-4 and MOG autoantibody discovery in idiopathic transverse myelitis epidemiology. Neurology. (2019) 93:e414–20. 10.1212/WNL.0000000000007828 31235660 PMC7508328

[6] Zalewski NL Flanagan EP Keegan BM. Evaluation of idiopathic transverse myelitis revealing specific myelopathy diagnoses. Neurology. (2018) 90:e96–e102. 10.1212/WNL.0000000000004796 29247071

[7] Flanagan EP Kaufmann TJ Krecke KN Aksamit AJ Pittock SJ Keegan BM. Discriminating long myelitis of neuromyelitis optica from sarcoidosis. Ann Neurol. (2016) 79:437–47. 10.1002/ana.24582 26677112

[8] Murphy OC Salazar-Camelo A Jimenez JA Barreras P Reyes MI Garcia MA. Clinical and MRI phenotypes of sarcoidosis-associated myelopathy. Neurol Neuroimmunol Neuroinflamm. (2020) 7:e722. 10.1212/NXI.0000000000000722 32269072 PMC7176244

[9] Flanagan EP McKeon A Lennon VA Kearns J Weinshenker BG Krecke KN. Paraneoplastic isolated myelopathy: clinical course and neuroimaging clues. Neurology. (2011) 76:2089–95. 10.1212/WNL.0b013e31821f468f 21670438

[10] Sechi E Morris PP McKeon A Pittock SJ Hinson SR Weinshenker BG. Glial fibrillary acidic protein IgG related myelitis: characterisation and comparison with aquaporin-4-IgG myelitis. J Neurol Neurosurg Psychiatry. (2019) 90:488–90. 10.1136/jnnp-2018-318004 30032117

[11] Guerra H Pittock SJ Moder KG Fryer JP Gadoth A Flanagan EP. Frequency of aquaporin-4 immunoglobulin G in longitudinally extensive transverse myelitis with antiphospholipid antibodies. Mayo Clin Proc. (2018) 93:1299–304. 10.1016/j.mayocp.2018.02.006 29655487

[12] Uygunoglu U Zeydan B Ozguler Y Ugurlu S Seyahi E Kocer N. Myelopathy in Behcet's disease: the Bagel sign. Ann Neurol. (2017) 82:288–98. 10.1002/ana.25004 28749553

[13] Sechi E Flanagan EP. Evaluation and management of acute myelopathy. Semin Neurol. (2021) 41:511–29. 10.1055/s-0041-1733792 34619778

[14] Mariano R Flanagan EP Weinshenker BG Palace J A. practical approach to the diagnosis of spinal cord lesions. Pract Neurol. (2018) 18:187–200. 10.1136/practneurol-2017-001845 29500319

[15] Transverse Myelitis Consortium Working G. Proposed diagnostic criteria and nosology of acute transverse myelitis. Neurology. (2002) 59:499–505. 10.1212/WNL.59.4.499 12236201

[16] Kitley JL Leite MI George JS Palace JA. The differential diagnosis of longitudinally extensive transverse myelitis. Mult Scler. (2012) 18:271–85. 10.1177/1352458511406165 21669935

[17] Savoldi F Nasr Z Hu W Schilaty ND Delgado AM Mandrekar J. McArdle sign: a specific sign of multiple sclerosis. Mayo Clin Proc. (2019) 94:1427–35. 10.1016/j.mayocp.2019.01.047 31303427

[18] Bejaoui K Rolak LA. What is the risk of permanent disability from a multiple sclerosis relapse? Neurology. (2010) 74:900–2. 10.1212/WNL.0b013e3181d55ee9 20231665

[19] Dubey D Pittock SJ Krecke KN Morris PP Sechi E Zalewski NL. Clinical, radiologic, and prognostic features of myelitis associated with myelin oligodendrocyte glycoprotein autoantibody. JAMA Neurol. (2019) 76:301–9. 10.1001/jamaneurol.2018.4053 30575890 PMC6440233

[20] Fadda G Alves CA O'Mahony J Castro DA Yeh EA Marrie RA. Comparison of spinal cord magnetic resonance imaging features among children with acquired demyelinating syndromes. JAMA Netw Open. (2021) 4:e2128871. 10.1001/jamanetworkopen.2021.28871 34643718 PMC8515204

[21] Bot JC Barkhof F Polman CH Lycklama a Nijeholt GJ de Groot V Bergers E. Spinal cord abnormalities in recently diagnosed MS patients: added value of spinal MRI examination. Neurology. (2004) 62:226–33. 10.1212/WNL.62.2.226 14745058

[22] Gajofatto A Benedetti MD. Prognostic factors of acute partial transverse myelitis. Arch Neurol. (2012) 69:1523; author reply-4. 10.1001/archneurol.2012.2302 23117920

[23] Murphy OC Mukharesh L Salazar-Camelo A Pardo CA Newsome SD. Early factors associated with later conversion to multiple sclerosis in patients presenting with isolated myelitis. J Neurol Neurosurg Psychiatry. (2021). 10.1136/jnnp-2020-325274. [Epub ahead of print]. 33687973

[24] Poullet Z Pique J Maarouf A Boutiere C Rico A Demortiere S. Pure relapsing short myelitis: part of the multiple sclerosis spectrum or new entity? Neurol Neuroimmunol Neuroinflamm. (2022) 9:1167. 10.1212/NXI.0000000000001167 35473885 PMC9128038

[25] Ayzenberg I Richter D Henke E Asseyer S Paul F Trebst C. Pain, depression, and quality of life in neuromyelitis optica spectrum disorder: a cross-sectional study of 166 AQP4 antibody-seropositive patients. Neurol Neuroimmunol Neuroinflamm. (2021) 8:e985. 10.1212/NXI.0000000000000985 34108267 PMC8058953

[26] Mariano R Messina S Kumar K Kuker W Leite MI Palace J. Comparison of clinical outcomes of transverse myelitis among adults with myelin oligodendrocyte glycoprotein antibody vs aquaporin-4 antibody disease. JAMA Netw Open. (2019) 2:e1912732. 10.1001/jamanetworkopen.2019.12732 31596489 PMC6802235

[27] Elsone L Townsend T Mutch K Das K Boggild M Nurmikko T. Neuropathic pruritus (itch) in neuromyelitis optica. Mult Scler. (2013) 19:475–9. 10.1177/1352458512457720 22936333

[28] Asseyer S Cooper G Paul F. Pain in NMOSD and MOGAD: a systematic literature review of pathophysiology, symptoms, and current treatment strategies. Front Neurol. (2020) 11:778. 10.3389/fneur.2020.00778 33473247 PMC7812141

[29] Sepulveda M Armangue T Martinez-Hernandez E Arrambide G Sola-Valls N Sabater L. Clinical spectrum associated with MOG autoimmunity in adults: significance of sharing rodent MOG epitopes. J Neurol. (2016) 263:1349–60. 10.1007/s00415-016-8147-7 27147513 PMC5831396

[30] Ciron J Cobo-Calvo A Audoin B Bourre B Brassat D Cohen M. Frequency and characteristics of short versus longitudinally extensive myelitis in adults with MOG antibodies: a retrospective multicentric study. Mult Scler. (2020) 26:936–44. 10.1177/1352458519849511 31148523

[31] Zhao-Fleming HH Valencia Sanchez C Sechi E Inbarasu J Wijdicks EF Pittock SJ. CNS demyelinating attacks requiring ventilatory support with myelin oligodendrocyte glycoprotein or aquaporin-4 antibodies. Neurology. (2021) 97:e1351–8. 10.1212/WNL.0000000000012599 34389648 PMC8480400

[32] Wingerchuk DM Hogancamp WF O'Brien PC Weinshenker BG. The clinical course of neuromyelitis optica (Devic's syndrome). Neurology. (1999) 53:1107–14. 10.1212/WNL.53.5.1107 10496275

[33] Kim SH Mealy MA Levy M Schmidt F Ruprecht K Paul F. Racial differences in neuromyelitis optica spectrum disorder. Neurology. (2018) 91:e2089–99. 10.1212/WNL.0000000000006574 30366977 PMC6282238

[34] Kitley J Waters P Woodhall M Leite MI Murchison A George J. Neuromyelitis optica spectrum disorders with aquaporin-4 and myelin-oligodendrocyte glycoprotein antibodies: a comparative study. JAMA Neurol. (2014) 71:276–83. 10.1001/jamaneurol.2013.5857 24425068

[35] Pittock SJ Weinshenker BG Lucchinetti CF Wingerchuk DM Corboy JR Lennon VA. Neuromyelitis optica brain lesions localized at sites of high aquaporin 4 expression. Arch Neurol. (2006) 63:964–8. 10.1001/archneur.63.7.964 16831965

[36] Sechi E Addis A Batzu L Mariotto S Ferrari S Conti M. Late presentation of NMOSD as rapidly progressive leukoencephalopathy with atypical clinical and radiological findings. Mult Scler. (2018) 24:685–8. 10.1177/1352458517721661 28814166

[37] Sechi E Cacciaguerra L Chen JJ Mariotto S Fadda G Dinoto A. Myelin oligodendrocyte glycoprotein antibody-associated disease (MOGAD): a review of clinical and MRI features, diagnosis, and management. Front Neurol. (2022) 13:885218. 10.3389/fneur.2022.885218 35785363 PMC9247462

[38] Cobo-Calvo A Ruiz A Rollot F Arrambide G Deschamps R Maillart E. Clinical features and risk of relapse in children and adults with myelin oligodendrocyte glycoprotein antibody-associated disease. Ann Neurol. (2021) 89:30–41. 10.1002/ana.25909 32959427

[39] de Mol CL Wong Y van Pelt ED Wokke B Siepman T Neuteboom RF. The clinical spectrum and incidence of anti-MOG-associated acquired demyelinating syndromes in children and adults. Mult Scler. (2020) 26:806–14. 10.1177/1352458519845112 31094288 PMC7294530

[40] Senanayake B Jitprapaikulsan J Aravinthan M Wijesekera JC Ranawaka UK Riffsy MT. Seroprevalence and clinical phenotype of MOG-IgG-associated disorders in Sri Lanka. J Neurol Neurosurg Psychiatry. (2019) 90:1381–3. 10.1136/jnnp-2018-320243 31387865 PMC6902071

[41] Baumann M Sahin K Lechner C Hennes EM Schanda K Mader S. Clinical and neuroradiological differences of paediatric acute disseminating encephalomyelitis with and without antibodies to the myelin oligodendrocyte glycoprotein. J Neurol Neurosurg Psychiatry. (2015) 86:265–72. 10.1136/jnnp-2014-308346 25121570

[42] Hyun JW Kwon YN Kim SM Lee HL Jeong WK Lee HJ. Value of area postrema syndrome in differentiating adults with AQP4 vs. MOG antibodies. Front Neurol. (2020) 11:396. 10.3389/fneur.2020.00396 32581992 PMC7287121

[43] Kunchok A Krecke KN Flanagan EP Jitprapaikulsan J Lopez-Chiriboga AS Chen JJ. Does area postrema syndrome occur in myelin oligodendrocyte glycoprotein-IgG-associated disorders (MOGAD)? Neurology. (2020) 94:85–8. 10.1212/WNL.0000000000008786 31827002

[44] Filippi M Preziosa P Banwell BL Barkhof F Ciccarelli O De Stefano N. Assessment of lesions on magnetic resonance imaging in multiple sclerosis: practical guidelines. Brain. (2019) 142:1858–75. 10.1093/brain/awz144 31209474 PMC6598631

[45] Asnafi S Morris PP Sechi E Pittock SJ Weinshenker BG Palace J. The frequency of longitudinally extensive transverse myelitis in MS: a population-based study. Mult Scler Relat Disord. (2020) 37:101487. 10.1016/j.msard.2019.101487 31707235

[46] Ciccarelli O Cohen JA Reingold SC Weinshenker BG International International Conference on Spinal Cord I Imaging in Multiple S. Spinal cord involvement in multiple sclerosis and neuromyelitis optica spectrum disorders. Lancet Neurol. (2019) 18:185–97. 10.1016/S1474-4422(18)30460-5 30663608

[47] Valsasina P Aboulwafa M Preziosa P Messina R Falini A Comi G. Cervical cord T1-weighted hypointense lesions at MR imaging in multiple sclerosis: relationship to cord atrophy and disability. Radiology. (2018) 288:234–44. 10.1148/radiol.2018172311 29664341

[48] Kearney H Altmann DR Samson RS Yiannakas MC Wheeler-Kingshott CA Ciccarelli O. Cervical cord lesion load is associated with disability independently from atrophy in MS. Neurology. (2015) 84:367–73. 10.1212/WNL.0000000000001186 25540312

[49] Klawiter EC Benzinger T Roy A Naismith RT Parks BJ Cross AH. Spinal cord ring enhancement in multiple sclerosis. Arch Neurol. (2010) 67:1395–8. 10.1001/archneurol.2010.271 21060017 PMC3057685

[50] Nakashima I Fujihara K Miyazawa I Misu T Narikawa K Nakamura M. Clinical and MRI features of Japanese patients with multiple sclerosis positive for NMO-IgG. J Neurol Neurosurg Psychiatry. (2006) 77:1073–5. 10.1136/jnnp.2005.080390 16505005 PMC2077753

[51] Fang W Zheng Y Yang F Cai MT Shen CH Liu ZR. Short segment myelitis as the initial and only manifestation of aquaporin-4 immunoglobulin G-positive neuromyelitis optica spectrum disorders. Ther Adv Neurol Disord. (2020) 13:1756286419898594. 10.1177/1756286419898594 32010226 PMC6971969

[52] Flanagan EP Weinshenker BG Krecke KN Lennon VA Lucchinetti CF McKeon A. Short myelitis lesions in aquaporin-4-IgG-positive neuromyelitis optica spectrum disorders. JAMA Neurol. (2015) 72:81–7. 10.1001/jamaneurol.2014.2137 25384099 PMC4552048

[53] Jarius S Ruprecht K Wildemann B Kuempfel T Ringelstein M Geis C. Contrasting disease patterns in seropositive and seronegative neuromyelitis optica: a multicentre study of 175 patients. J Neuroinflammation. (2012) 9:14. 10.1186/1742-2094-9-14 22260418 PMC3283476

[54] Kim HJ Paul F Lana-Peixoto MA Tenembaum S Asgari N Palace J. MRI characteristics of neuromyelitis optica spectrum disorder: an international update. Neurology. (2015) 84:1165–73. 10.1212/WNL.0000000000001367 25695963 PMC4371410

[55] Iorio R Damato V Mirabella M Evoli A Marti A Plantone D. Distinctive clinical and neuroimaging characteristics of longitudinally extensive transverse myelitis associated with aquaporin-4 autoantibodies. J Neurol. (2013) 260:2396–402. 10.1007/s00415-013-6997-9 23793787

[56] Yonezu T Ito S Mori M Ogawa Y Makino T Uzawa A. “Bright spotty lesions” on spinal magnetic resonance imaging differentiate neuromyelitis optica from multiple sclerosis. Mult Scler. (2014) 20:331–7. 10.1177/1352458513495581 23828869

[57] Hyun JW Lee HL Park J Kim J Min JH Kim BJ. Brighter spotty lesions on spinal MRI help differentiate AQP4 antibody-positive NMOSD from MOGAD. Mult Scler. (2022) 28:989–92. 10.1177/13524585211060326 34865555

[58] Rabaste S Cobo-Calvo A Nistiriuc-Muntean V Vukusic S Marignier R Cotton F. Diagnostic value of bright spotty lesions on MRI after a first episode of acute myelopathy. J Neuroradiol. (2021) 48:28–36. 10.1016/j.neurad.2020.04.006 32407908

[59] Sechi E Krecke KN Pittock SJ Dubey D Lopez-Chiriboga AS Kunchok A. Frequency and characteristics of MRI-negative myelitis associated with MOG autoantibodies. Mult Scler. (2020) 2020:1352458520907900. 10.1177/1352458520907900 32103708 PMC7500857

[60] Rinaldi S Davies A Fehmi J Beadnall HN Wang J Hardy TA. Overlapping central and peripheral nervous system syndromes in MOG antibody-associated disorders. Neurol Neuroimmunol Neuroinflamm. (2021) 8:e924. 10.1212/NXI.0000000000000924 33272955 PMC7803332

[61] Mohseni SH Skejoe HPB Wuerfel J Paul F Reindl M Jarius S. Leptomeningeal and intraparenchymal blood barrier disruption in a MOG-IgG-positive patient. Case Rep Neurol Med. (2018) 2018:1365175. 10.1155/2018/1365175 30834146 PMC6374805

[62] Dinoto A Sechi E Flanagan EP Ferrari S Solla P Mariotto S. Serum and cerebrospinal fluid biomarkers in neuromyelitis optica spectrum disorder and myelin oligodendrocyte glycoprotein associated disease. Front Neurol. (2022) 13:866824. 10.3389/fneur.2022.866824 35401423 PMC8983882

[63] Deisenhammer F Zetterberg H Fitzner B Zettl UK. The cerebrospinal fluid in multiple sclerosis. Front Immunol. (2019) 10:726. 10.3389/fimmu.2019.00726 31031747 PMC6473053

[64] Chabas D Ness J Belman A Yeh EA Kuntz N Gorman MP. Younger children with MS have a distinct CSF inflammatory profile at disease onset. Neurology. (2010) 74:399–405. 10.1212/WNL.0b013e3181ce5db0 20124205 PMC2816008

[65] Fadda G Waters P Woodhall M Brown RA O'Mahony J Castro DA. Serum MOG-IgG in children meeting multiple sclerosis diagnostic criteria. Mult Scler. (2022) 2022:13524585221093789. 10.1177/13524585221093789 35581944 PMC9442635

[66] Jarius S Lechner C Wendel EM Baumann M Breu M Schimmel M. Cerebrospinal fluid findings in patients with myelin oligodendrocyte glycoprotein (MOG) antibodies. Part 2: Results from 108 lumbar punctures in 80 pediatric patients. J Neuroinflam. (2020) 17:262. 10.1186/s12974-020-01825-1 32883358 PMC7470445

[67] Sechi E Buciuc M Flanagan EP Pittock SJ Banks SA Lopez-Chiriboga AS. Variability of cerebrospinal fluid findings by attack phenotype in myelin oligodendrocyte glycoprotein-IgG-associated disorder. Mult Scler Relat Disord. (2020) 47:102638. 10.1016/j.msard.2020.102638 33276239

[68] Defresne P Hollenberg H Husson B Tabarki B Landrieu P Huault G. Acute transverse myelitis in children: clinical course and prognostic factors. J Child Neurol. (2003) 18:401–6. 10.1177/08830738030180060601 12886975

[69] West T Wyatt M High A Bostrom A Waubant E. Are initial demyelinating event recovery and time to second event under differential control? Neurology. (2006) 67:809–13. 10.1212/01.wnl.0000234031.30756.a0 16966542

[70] Lublin FD Baier M Cutter G. Effect of relapses on development of residual deficit in multiple sclerosis. Neurology. (2003) 61:1528–32. 10.1212/01.WNL.0000096175.39831.21 14663037

[71] Leone MA Bonissoni S Collimedaglia L Tesser F Calzoni S Stecco A. Factors predicting incomplete recovery from relapses in multiple sclerosis: a prospective study. Mult Scler. (2008) 14:485–93. 10.1177/1352458507084650 18208889

[72] Kantarci OH Zeydan B Atkinson EJ Conway BL Castrillo-Viguera C Rodriguez M. Relapse recovery: the forgotten variable in multiple sclerosis clinical trials. Neurol Neuroimmunol Neuroinflamm. (2020) 7:e653. 10.1212/NXI.0000000000000653 31848231 PMC6943367

[73] Novotna M Paz Soldan MM Abou Zeid N Kale N Tutuncu M Crusan DJ. Poor early relapse recovery affects onset of progressive disease course in multiple sclerosis. Neurology. (2015) 85:722–9. 10.1212/WNL.0000000000001856 26208962 PMC4553030

[74] Beck RW Cleary PA Anderson MM Jr Keltner JL Shults WT Kaufman DI. A randomized, controlled trial of corticosteroids in the treatment of acute optic neuritis: the Optic Neuritis Study Group. N Engl J Med. (1992) 326:581–8. 10.1056/NEJM199202273260901 1734247

[75] Conway BL Zeydan B Uygunoglu U Novotna M Siva A Pittock SJ. Age is a critical determinant in recovery from multiple sclerosis relapses. Mult Scler. (2019) 25:1754–63. 10.1177/1352458518800815 30303037

[76] Sotiropoulos MG Lokhande H Healy BC Polgar-Turcsanyi M Glanz BI Bakshi R. Relapse recovery in multiple sclerosis: effect of treatment and contribution to long-term disability. Mult Scler J Exp Transl Clin. (2021) 7:20552173211015503. 10.1177/20552173211015503 34104471 PMC8165535

[77] Menascu S Khavkin Y Zilkha-Falb R Dolev M Magalashvili D Achiron A. Clinical and transcriptional recovery profiles in pediatric and adult multiple sclerosis patients. Ann Clin Transl Neurol. (2021) 8:81–94. 10.1002/acn3.51244 33197148 PMC7818128

[78] Banerjee A Ng J Coleman J Ospina JP Mealy M Levy M. Outcomes from acute attacks of neuromyelitis optica spectrum disorder correlate with severity of attack, age and delay to treatment. Mult Scler Relat Disord. (2019) 28:60–3. 10.1016/j.msard.2018.12.010 30554039 PMC6397696

[79] Papathanasiou A Tanasescu R Tench CR Rocha MF Bose S Constantinescu CS. Age at onset predicts outcome in aquaporin-4-IgG positive neuromyelitis optica spectrum disorder from a United Kingdom population. J Neurol Sci. (2021) 431:120039. 10.1016/j.jns.2021.120039 34715481

[80] Camera V Messina S Elhadd KT Sanpera-Iglesias J Mariano R Hacohen Y. Early predictors of disability of paediatric-onset AQP4-IgG-seropositive neuromyelitis optica spectrum disorders. J Neurol Neurosurg Psychiatry. (2022) 93:101–11. 10.1136/jnnp-2021-327206 34583946

[81] Mealy MA Mossburg SE Kim SH Messina S Borisow N Lopez-Gonzalez R. Long-term disability in neuromyelitis optica spectrum disorder with a history of myelitis is associated with age at onset, delay in diagnosis/preventive treatment, MRI lesion length and presence of symptomatic brain lesions. Mult Scler Relat Disord. (2019) 28:64–8. 10.1016/j.msard.2018.12.011 30554040 PMC6397677

[82] Takano R Misu T Takahashi T Sato S Fujihara K Itoyama Y. Astrocytic damage is far more severe than demyelination in NMO: a clinical CSF biomarker study. Neurology. (2010) 75:208–16. 10.1212/WNL.0b013e3181e2414b 20644148

[83] Palace J Lin DY Zeng D Majed M Elsone L Hamid S. Outcome prediction models in AQP4-IgG positive neuromyelitis optica spectrum disorders. Brain. (2019) 142:1310–23. 10.1093/brain/awz054 30938427 PMC6487334

[84] Mealy MA Kessler RA Rimler Z Reid A Totonis L Cutter G. Mortality in neuromyelitis optica is strongly associated with African ancestry. Neurol Neuroimmunol Neuroinflamm. (2018) 5:e468. 10.1212/NXI.0000000000000468 29892608 PMC5994702

[85] Kleiter I Gahlen A Borisow N Fischer K Wernecke KD Wegner B. Neuromyelitis optica: Evaluation of 871 attacks and 1,153 treatment courses. Ann Neurol. (2016) 79:206–16. 10.1002/ana.24554 26537743

[86] Bonnan M Valentino R Debeugny S Merle H Ferge JL Mehdaoui H. Short delay to initiate plasma exchange is the strongest predictor of outcome in severe attacks of NMO spectrum disorders. J Neurol Neurosurg Psychiatry. (2018) 89:346–51. 10.1136/jnnp-2017-316286 29030418

[87] Jurynczyk M Messina S Woodhall MR Raza N Everett R Roca-Fernandez A. Clinical presentation and prognosis in MOG-antibody disease: a UK study. Brain. (2017) 140:3128–38. 10.1093/brain/awx276 29136091

[88] Rovaris M Confavreux C Furlan R Kappos L Comi G Filippi M. Secondary progressive multiple sclerosis: current knowledge and future challenges. Lancet Neurol. (2006) 5:343–54. 10.1016/S1474-4422(06)70410-0 16545751

[89] Fadda G Armangue T Hacohen Y Chitnis T Banwell B. Paediatric multiple sclerosis and antibody-associated demyelination: clinical, imaging, and biological considerations for diagnosis and care. Lancet Neurol. (2021) 20:136–49. 10.1016/S1474-4422(20)30432-4 33484648

[90] Ransohoff RM Hafler DA Lucchinetti CF. Multiple sclerosis—a quiet revolution. Nat Rev Neurol. (2015) 11:134–42. 10.1038/nrneurol.2015.14 25686758 PMC4556342

[91] Compston A Coles A. Multiple sclerosis. Lancet. (2008) 372:1502–17. 10.1016/S0140-6736(08)61620-7 18970977

[92] Keegan BM Kaufmann TJ Weinshenker BG Kantarci OH Schmalstieg WF Paz Soldan MM. Progressive solitary sclerosis: Gradual motor impairment from a single CNS demyelinating lesion. Neurology. (2016) 87:1713–9. 10.1212/WNL.0000000000003235 27638926 PMC5085075

[93] Schmalstieg WF Keegan BM Weinshenker BG. Solitary sclerosis: progressive myelopathy from solitary demyelinating lesion. Neurology. (2012) 78:540–4. 10.1212/WNL.0b013e318247cc8c 22323754 PMC12435764

[94] Thompson AJ Banwell BL Barkhof F Carroll WM Coetzee T Comi G. Diagnosis of multiple sclerosis: 2017 revisions of the McDonald criteria. Lancet Neurol. (2018) 17:162–73. 10.1016/S1474-4422(17)30470-2 29275977

[95] Keegan BM Kaufmann TJ Weinshenker BG Kantarci OH Schmalstieg WF Paz Soldan MM. Progressive motor impairment from a critically located lesion in highly restricted CNS-demyelinating disease. Mult Scler. (2018) 24:1445–52. 10.1177/1352458518781979 30047830

[96] Sechi E Keegan BM Kaufmann TJ Kantarci OH Weinshenker BG Flanagan EP. Unilateral motor progression in MS: association with a critical corticospinal tract lesion. Neurology. (2019) 93:e628–e34. 10.1212/WNL.0000000000007944 31289142

[97] Sechi E Messina S Keegan BM Buciuc M Pittock SJ Kantarci OH. Critical spinal cord lesions associate with secondary progressive motor impairment in long-standing MS: a population-based case-control study. Mult Scler. (2021) 27:667–73. 10.1177/1352458520929192 32552535 PMC10477711

[98] Barakat B Messina S Nayak S Kassa R Sechi E Flanagan EP. Cerebrospinal fluid evaluation in patients with progressive motor impairment due to critical central nervous system demyelinating lesions. Mult Scler J Exp Transl Clin. (2022) 8:20552173211052159. 10.1177/20552173211052159 35047187 PMC8761886

[99] Kassa RM Sechi E Flanagan EP Kaufmann TJ Kantarci OH Weinshenker BG. Onset of progressive motor impairment in patients with critical central nervous system demyelinating lesions. Mult Scler. (2021) 27:895–902. 10.1177/1352458520940983 32667237

[100] Nayak S Sechi E Flanagan EP Messina S Kassa R Kantarci O. Inflammatory activity following motor progression due to critical CNS demyelinating lesions. Mult Scler. (2021) 27:1037–45. 10.1177/1352458520948745 32812487

[101] Kantarci OH Lebrun C Siva A Keegan MB Azevedo CJ Inglese M. Primary progressive multiple sclerosis evolving from radiologically isolated syndrome. Ann Neurol. (2016) 79:288–94. 10.1002/ana.24564 26599831

[102] Weinshenker BG Wingerchuk DM Vukusic S Linbo L Pittock SJ Lucchinetti CF. Neuromyelitis optica IgG predicts relapse after longitudinally extensive transverse myelitis. Ann Neurol. (2006) 59:566–9. 10.1002/ana.20770 16453327

[103] Kunchok A Malpas C Nytrova P Havrdova EK Alroughani R Terzi M. Clinical and therapeutic predictors of disease outcomes in AQP4-IgG + neuromyelitis optica spectrum disorder. Mult Scler Relat Disord. (2020) 38:101868. 10.1016/j.msard.2019.101868 31877445

[104] Stellmann JP Krumbholz M Friede T Gahlen A Borisow N Fischer K. Immunotherapies in neuromyelitis optica spectrum disorder: efficacy and predictors of response. J Neurol Neurosurg Psychiatry. (2017) 88:639–47. 10.1136/jnnp-2017-315603 28572277 PMC5537514

[105] Jiao Y Fryer JP Lennon VA McKeon A Jenkins SM Smith CY. Aquaporin 4 IgG serostatus and outcome in recurrent longitudinally extensive transverse myelitis. JAMA Neurol. (2014) 71:48–54. 10.1001/jamaneurol.2013.5055 24248262 PMC3934000

[106] Jiao Y Fryer JP Lennon VA Jenkins SM Quek AM Smith CY. Updated estimate of AQP4-IgG serostatus and disability outcome in neuromyelitis optica. Neurology. (2013) 81:1197–204. 10.1212/WNL.0b013e3182a6cb5c 23997151 PMC3795610

[107] Wingerchuk DM Banwell B Bennett JL Cabre P Carroll W Chitnis T. International consensus diagnostic criteria for neuromyelitis optica spectrum disorders. Neurology. (2015) 85:177–89. 10.1212/WNL.0000000000001729 26092914 PMC4515040

[108] Kim SH Jang H Park NY Kim Y Kim SY Lee MY. Discontinuation of immunosuppressive therapy in patients with neuromyelitis optica spectrum disorder with aquaporin-4 antibodies. Neurol Neuroimmunol Neuroinflamm. (2021) 8:e947. 10.1212/NXI.0000000000000947 33622675 PMC7903808

[109] Waters P Fadda G Woodhall M O'Mahony J Brown RA Castro DA. Serial anti-myelin oligodendrocyte glycoprotein antibody analyses and outcomes in children with demyelinating syndromes. JAMA Neurol. (2020) 77:82–93. 10.1001/jamaneurol.2019.2940 31545352 PMC6763982

[110] Marignier R Hacohen Y Cobo-Calvo A Probstel AK Aktas O Alexopoulos H. Myelin-oligodendrocyte glycoprotein antibody-associated disease. Lancet Neurol. (2021) 20:762–72. 10.1016/S1474-4422(21)00218-0 34418402

[111] Akaishi T Misu T Fujihara K Takahashi T Takai Y Nishiyama S. Relapse activity in the chronic phase of anti-myelin-oligodendrocyte glycoprotein antibody-associated disease. J Neurol. (2021) 269:3136–46. 10.1007/s00415-021-10914-x 34820735 PMC9120114

[112] Clarke L Arnett S Bukhari W Khalilidehkordi E Jimenez Sanchez S O'Gorman C. MRI patterns distinguish AQP4 antibody positive neuromyelitis optica spectrum disorder from multiple sclerosis. Front Neurol. (2021) 12:722237. 10.3389/fneur.2021.722237 34566866 PMC8458658

[113] Lopez-Chiriboga AS Majed M Fryer J Dubey D McKeon A Flanagan EP. Association of MOG-IgG serostatus with relapse after acute disseminated encephalomyelitis and proposed diagnostic criteria for MOG-IgG-associated disorders. JAMA Neurol. (2018) 75:1355–63. 10.1001/jamaneurol.2018.1814 30014148 PMC6248120

[114] Satukijchai C Mariano R Messina S Sa M Woodhall MR Robertson NP. Factors associated with relapse and treatment of myelin oligodendrocyte glycoprotein antibody-associated disease in the United Kingdom. JAMA Netw Open. (2022) 5:e2142780. 10.1001/jamanetworkopen.2021.42780 35006246 PMC8749481

[115] Deschamps R Pique J Ayrignac X Collongues N Audoin B Zephir H. The long-term outcome of MOGAD: an observational national cohort study of 61 patients. Eur J Neurol. (2021) 28:1659–64. 10.1111/ene.14746 33528851

[116] Jitprapaikulsan J Lopez Chiriboga AS Flanagan EP Fryer JP McKeon A Weinshenker BG. Novel glial targets and recurrent longitudinally extensive transverse myelitis. JAMA Neurol. (2018) 75:892–5. 10.1001/jamaneurol.2018.0805 29710213 PMC5933459

[117] Akaishi T Misu T Takahashi T Takai Y Nishiyama S Fujimori J. Progression pattern of neurological disability with respect to clinical attacks in anti-MOG antibody-associated disorders. J Neuroimmunol. (2020) 351:577467. 10.1016/j.jneuroim.2020.577467 33388541

[118] Held F Kalluri SR Berthele A Klein AK Reindl M Hemmer B. Frequency of myelin oligodendrocyte glycoprotein antibodies in a large cohort of neurological patients. Mult Scler J Exp Transl Clin. (2021) 7:20552173211022767. 10.1177/20552173211022767 34262784 PMC8246507

[119] Zara P Floris V Flanagan EP Lopez-Chiriboga AS Weinshenker BG Solla P. Clinical significance of myelin oligodendrocyte glycoprotein autoantibodies in patients with typical MS lesions on MRI. Mult Scler J Exp Transl Clin. (2021) 7:20552173211048761. 10.1177/20552173211048761 34820135 PMC8606934

[120] Sechi E Krecke KN Messina SA Buciuc M Pittock SJ Chen JJ. Comparison of MRI lesion evolution in different central nervous system demyelinating disorders. Neurology. (2021) 97:e1097–109. 10.1212/WNL.0000000000012467 34261784 PMC8456356

[121] Asgari N Skejoe HP Lillevang ST Steenstrup T Stenager E Kyvik KO. Modifications of longitudinally extensive transverse myelitis and brainstem lesions in the course of neuromyelitis optica (NMO): a population-based, descriptive study. BMC Neurol. (2013) 13:33. 10.1186/1471-2377-13-33 23566260 PMC3622587

[122] Zecca C Disanto G Sormani MP Riccitelli GC Cianfoni A Del Grande F. Relevance of asymptomatic spinal MRI lesions in patients with multiple sclerosis. Mult Scler. (2016) 22:782–91. 10.1177/1352458515599246 26459149

[123] Camera V Holm-Mercer L Ali AAH Messina S Horvat T Kuker W. Frequency of new silent MRI lesions in myelin oligodendrocyte glycoprotein antibody disease and aquaporin-4 antibody neuromyelitis optica spectrum disorder. JAMA Netw Open. (2021) 4:e2137833. 10.1001/jamanetworkopen.2021.37833 34878547 PMC8655599

[124] Cacciaguerra L Valsasina P Mesaros S Martinelli V Drulovic J Filippi M. Spinal cord atrophy in neuromyelitis optica spectrum disorders is spatially related to cord lesions and disability. Radiology. (2020) 297:154–63. 10.1148/radiol.2020192664 32720869

[125] Chien C Scheel M Schmitz-Hubsch T Borisow N Ruprecht K Bellmann-Strobl J. Spinal cord lesions and atrophy in NMOSD with AQP4-IgG and MOG-IgG associated autoimmunity. Mult Scler. (2019) 25:1926–36. 10.1177/1352458518815596 30475082

[126] Mariano R Messina S Roca-Fernandez A Leite MI Kong Y Palace JA. Quantitative spinal cord MRI in MOG-antibody disease, neuromyelitis optica and multiple sclerosis. Brain. (2021) 144:198–212. 10.1093/brain/awaa347 33206944

[127] Tremlett H Paty D Devonshire V. Disability progression in multiple sclerosis is slower than previously reported. Neurology. (2006) 66:172–7. 10.1212/01.wnl.0000194259.90286.fe 16434648

[128] Runmarker B Andersen O. Prognostic factors in a multiple sclerosis incidence cohort with twenty-five years of follow-up. Brain. (1993) 116 (Pt 1):117–34. 10.1093/brain/116.1.117 8453453

[129] Pittock SJ McClelland RL Mayr WT Jorgensen NW Weinshenker BG Noseworthy J. Clinical implications of benign multiple sclerosis: a 20-year population-based follow-up study. Ann Neurol. (2004) 56:303–6. 10.1002/ana.20197 15293286

[130] Menon S Shirani A Zhao Y Oger J Traboulsee A Freedman MS. Characterising aggressive multiple sclerosis. J Neurol Neurosurg Psychiatry. (2013) 84:1192–8. 10.1136/jnnp-2013-304951 23744892

[131] Gholipour T Healy B Baruch NF Weiner HL Chitnis T. Demographic and clinical characteristics of malignant multiple sclerosis. Neurology. (2011) 76:1996–2001. 10.1212/WNL.0b013e31821e559d 21646626

[132] Ellenberger D Flachenecker P Haas J Hellwig K Paul F Stahmann A. Is benign MS really benign? What a meaningful classification beyond the EDSS must take into consideration. Mult Scler Relat Disord. (2020) 46:102485. 10.1016/j.msard.2020.102485 32980646

[133] Langer-Gould A Popat RA Huang SM Cobb K Fontoura P Gould MK. Clinical and demographic predictors of long-term disability in patients with relapsing-remitting multiple sclerosis: a systematic review. Arch Neurol. (2006) 63:1686–91. 10.1001/archneur.63.12.1686 17172607

[134] Sombekke MH Wattjes MP Balk LJ Nielsen JM Vrenken H Uitdehaag BM. Spinal cord lesions in patients with clinically isolated syndrome: a powerful tool in diagnosis and prognosis. Neurology. (2013) 80:69–75. 10.1212/WNL.0b013e31827b1a67 23243070

[135] Brownlee WJ Altmann DR Alves Da Mota P Swanton JK Miszkiel KA Wheeler-Kingshott CG. Association of asymptomatic spinal cord lesions and atrophy with disability 5 years after a clinically isolated syndrome. Mult Scler. (2017) 23:665–74. 10.1177/1352458516663034 27481210

[136] Okuda DT Mowry EM Cree BA Crabtree EC Goodin DS Waubant E. Asymptomatic spinal cord lesions predict disease progression in radiologically isolated syndrome. Neurology. (2011) 76:686–92. 10.1212/WNL.0b013e31820d8b1d 21270417 PMC3053327

[137] Bischof A Papinutto N Keshavan A Rajesh A Kirkish G Zhang X. Spinal cord atrophy predicts progressive disease in relapsing multiple sclerosis. Ann Neurol. (2022) 91:268–81. 10.1002/ana.26281 34878197 PMC8916838

[138] Tsagkas C Magon S Gaetano L Pezold S Naegelin Y Amann M. Spinal cord volume loss: a marker of disease progression in multiple sclerosis. Neurology. (2018) 91:e349–e58. 10.1212/WNL.0000000000005853 29950437

[139] Schlaeger R Papinutto N Zhu AH Lobach IV Bevan CJ Bucci M. Association between thoracic spinal cord gray matter atrophy and disability in multiple sclerosis. JAMA Neurol. (2015) 72:897–904. 10.1001/jamaneurol.2015.0993 26053119 PMC6002864

[140] Bernitsas E Bao F Seraji-Bozorgzad N Chorostecki J Santiago C Tselis A. Spinal cord atrophy in multiple sclerosis and relationship with disability across clinical phenotypes. Mult Scler Relat Disord. (2015) 4:47–51. 10.1016/j.msard.2014.11.002 25787052

